# Multisensory Rhythmic Entrainment as a Mechanistic Framework for Modulating Prefrontal Network Stability in Focal Epilepsy

**DOI:** 10.3390/brainsci15121318

**Published:** 2025-12-10

**Authors:** Ekaterina Andreevna Narodova

**Affiliations:** Department of Neurology, Prof. V.F. Voyno-Yasenetsky Krasnoyarsk State Medical University, Krasnoyarsk 660022, Russia; katya_n2001@mail.ru; Tel.: +7-902-924-89-95

**Keywords:** epilepsy, drug-resistant epilepsy, neural entrainment, multisensory stimulation, interhemispheric coherence, prefrontal control networks, digital neuromodulation

## Abstract

Epilepsy is increasingly conceptualized as a disorder of large-scale network instability, involving impairments in interhemispheric connectivity, prefrontal inhibitory control, and slow-frequency temporal processing. Rhythmic sensory stimulation—auditory, vibrotactile, or multisensory—can entrain neuronal oscillations and modulate attentional and sensorimotor networks, yet its mechanistic relevance to epileptic network physiology remains insufficiently explored. This conceptual and mechanistic article integrates empirical findings from entrainment research, prefrontal timing theories, multisensory integration, and network-based models of seizure dynamics and uses them to formulate a hypothesis-driven framework for multisensory exogenous rhythmic stimulation (ERS) in focal epilepsy. Rather than presenting a tested intervention, we propose a set of speculative mechanistic pathways through which low-frequency rhythmic cues might serve as an external temporal reference, engage fronto-parietal control systems, facilitate multisensory-driven sensorimotor coupling, and potentially modulate interhemispheric frontal coherence. These putative mechanisms are illustrated by exploratory neurophysiological observations, including a small pilot study reporting frontal coherence changes during mobile ERS exposure, but they have not yet been validated in controlled experimental settings. The framework does not imply therapeutic benefit; instead, it identifies theoretical pathways through which rhythmic sensory cues may transiently interact with epileptic networks. The proposed model is intended as a conceptual foundation for future neurophysiological validation, computational simulations, and early feasibility research in the emerging field of digital neuromodulation, rather than as evidence of clinical efficacy. This Hypothesis article formulates explicitly testable predictions regarding how multisensory ERS may transiently modulate candidate physiological markers of prefrontal network stability in focal epilepsy.

## 1. Introduction

Epilepsy is one of the most common chronic, disabling neurological disorders, affecting approximately 50 million people worldwide [1]. In many cases, onset occurs in childhood or early adulthood, and patients may carry the disease throughout life as a substantial physical, psychological, and social burden. Consequently, the role of the epileptologist extends beyond selecting antiseizure medications (ASMs) and includes educating patients to live with the disorder, supporting adaptation, and mitigating both external and internalized stigma. Against this background, health-related quality of life becomes a central treatment objective for people with epilepsy. Unfortunately, despite continuous expansion of the ASM armamentarium, about one-third of patients continue to experience seizures even under adequate pharmacotherapy, constituting the population with drug-resistant epilepsy (DRE) [2]. The limited effectiveness of medication alone has intensified interest in additional non-pharmacological approaches capable of modulating cortical excitability or large-scale network synchronization.

In recent years, rhythmic sensory stimulation—auditory, vibrotactile, or multisensory—has attracted growing attention as a promising neuromodulatory strategy. Neural entrainment refers to the phase alignment of endogenous brain oscillations to an external periodic stimulus and has been robustly demonstrated in auditory, somatosensory, and visual modalities [3,4,5]. Auditory rhythmic stimulation can induce frequency-specific changes in cortical network activity, particularly within prefrontal and temporo-parietal regions, influencing attention, emotional regulation, and sensorimotor coupling [6,7]. Vibrotactile rhythmic stimulation similarly evokes phase-locked responses in sensorimotor and frontal cortices, indicating related resonance mechanisms [8].

In clinical neurology, rhythmic sensory stimulation has been extensively investigated across several conditions. Rhythmic auditory stimulation has been shown to improve gait and motor timing in Parkinson’s disease [9] and is employed in post-stroke motor rehabilitation [10]. Auditory and vibrotactile rhythms have also been explored as tools to modulate sleep architecture [11], reduce anxiety symptoms [12], and influence pain perception via oscillatory mechanisms [13]. These findings support the notion that external rhythmic signals can interact with large-scale neural networks in clinically meaningful ways. However, most of this mechanistic and clinical evidence comes from non-epilepsy populations, such as Parkinson’s disease, stroke rehabilitation, sleep modulation, anxiety, or pain. In these conditions, the targeted circuits, symptom dimensions, and risk–benefit considerations differ substantially from ictogenic networks in epilepsy. Therefore, extrapolations to epilepsy in the present article are intentionally conservative and focus on general principles of neural entrainment and large-scale network control rather than on disease-specific therapeutic effects.

In epileptology, rhythmic stimulation has been studied predominantly in the context of invasive neuromodulation—vagus nerve stimulation (VNS), deep brain stimulation (DBS), and responsive neurostimulation (RNS)—all of which employ periodic or adaptive stimulation paradigms to modulate epileptic networks [14,15]. Non-invasive rhythmic sensory interventions in epilepsy remain far less explored. This relative underrepresentation likely reflects several factors. Historically, research priorities in epilepsy neuromodulation have focused on invasive approaches (VNS, DBS, RNS) with direct access to ictogenic hubs, whereas sensory stimulation has been perceived as more indirect and potentially less potent. In addition, concerns about inadvertently provoking seizures with patterned sensory input, the low base rate and unpredictability of ictal events, and methodological challenges in capturing seizure-related outcomes in small samples have all contributed to the slow accumulation of evidence. Several preliminary studies have reported that auditory or somatosensory rhythmic cues can alter EEG rhythmicity, modulate interhemispheric coherence, or influence prefrontal network synchronization in small patient cohorts [16,17,18]. However, these data are exploratory, and their clinical implications for seizure modulation are not yet established.

Within the broader field of digital medicine, mobile applications are emerging as a means to deliver rhythmic stimuli in everyday environments. Such tools are being investigated as supports for self-regulation, stress reduction, and attentional control in chronic neurological conditions [19]. Exogenous rhythmic stimulation (ERS) implemented via mobile devices could, in principle, interact with cortical networks without the need for implanted hardware. A pilot study of the EpiTapp^®^ mobile application demonstrated an increase in interhemispheric coherence in frontal regions in patients with focal epilepsy during rhythmic sensory stimulation [20]. These findings are preliminary, require independent replication, and do not permit any inference regarding clinical efficacy at this stage.

In this article, we use the term “digital neuromodulation” to refer to non-invasive modulation of neural dynamics by sensory or other stimuli delivered through consumer digital devices (e.g., smartphones, tablets, and wearables). The current framework focuses on open-loop rhythmic sensory stimulation, while acknowledging that future implementations may incorporate closed-loop adaptations based on physiological or behavioral signals.

The aim of the present work is to develop a mechanistic and conceptual model of how auditory and vibrotactile rhythmic stimulation, delivered through mobile technologies, may interact with brain networks relevant to epilepsy. We integrate current knowledge on neural entrainment, sensory-driven synchronization, epileptic network physiology, and digital neuromodulation, and propose a hypothesis-driven framework that requires subsequent experimental testing. This article does not present clinical effectiveness data; rather, it provides a theoretical foundation and outlines future research directions necessary to evaluate the translational potential of this approach.

We specifically hypothesize that low-frequency (~1 Hz) multisensory exogenous rhythmic stimulation (ERS) delivered via mobile technologies can transiently enhance interhemispheric frontal coherence and stabilize prefrontal timing networks in focal epilepsy as a mechanistic target. In the present Hypothesis Article, these changes are treated strictly as short-term physiological markers of network organization, without advancing any claims regarding clinical efficacy or seizure control.

## 2. Literature Basis and Conceptual Approach

### 2.1. Type of Article

This work is a conceptual and mechanistic review, integrating findings from neurophysiology, sensory entrainment research, digital neuromodulation, and epilepsy network science to develop a hypothesis-driven framework of multisensory exogenous rhythmic stimulation (ERS) in focal epilepsy. The article does not evaluate clinical outcomes and does not present interventional or trial data.

### 2.2. Literature Search Strategy

A narrative literature search was conducted using PubMed and Scopus, supplemented by targeted searches in Google Scholar, covering publications from January 2015 to November 2025. Search terms included combinations of: “epilepsy”, “ictogenic networks”, “entrainment”, “rhythmic sensory stimulation”, “auditory stimulation”, “vibrotactile stimulation”, “multisensory integration”,” interhemispheric coherence”, “prefrontal cortex”, “temporal processing”, “digital neuromodulation”, “mobile health”, “neuromodulation technologies”.

We prioritized: peer-reviewed clinical neurophysiology studies, mechanistic and computational neuroscience research, entrainment studies in auditory/somatosensory modalities, neuromodulation literature (non-invasive and invasive), and digital health implementations involving rhythmic cues.

### 2.3. Scope of Evidence Considered

We focused on studies that addressed sensory entrainment (auditory, tactile, multisensory), temporal processing networks, prefrontal timing, interhemispheric coherence, and mechanistic models of ictogenic networks. We did not consider papers that lacked mechanistic or neurophysiological content, focused on purely non-neurological sensory stimulation, or reported therapeutic effects without physiological evidence.

### 2.4. Data Synthesis

Given the conceptual nature of the work, no quantitative synthesis, meta-analysis, or risk-of-bias assessment was performed. Evidence was integrated qualitatively and organized into mechanistic domains: (1) neural timing and prefrontal control, (2) temporal dysregulation in epilepsy, (3) multisensory rhythmic entrainment, and (4) network-based hypotheses for ERS interaction with epileptic circuits.

### 2.5. Ethical Compliance

This article does not involve new studies with human participants or animals. No new data were generated or collected for the purposes of this work. The EEG findings summarized in Section 5.3 refer to previously conducted exploratory research that has been reported elsewhere [20] and are described here only in aggregate, without any individual-level data.

### 2.6. Distinguishing Empirical Evidence from Conceptual Hypotheses

Given the mixed empirical and conceptual nature of this work, it is important to clearly distinguish between evidence-supported statements and speculative elements of the framework. Throughout the manuscript, empirical findings are restricted to previously published data on (I) large-scale network abnormalities in focal epilepsy, (II) general principles of sensory entrainment and multisensory integration, and (III) exploratory EEG observations of frontal coherence changes during mobile ERS exposure [20]. These data provide a qualitative background and illustration of the proposed ideas but do not establish causal relationships between ERS and ictogenic processes.

By contrast, all claims regarding “network stabilization”, potential modulation of seizure propagation thresholds, and the broader translational implications of mobile ERS are treated as hypotheses. They are formulated as putative mechanistic pathways that might link external rhythmic cues to prefrontal and interhemispheric network dynamics under specific conditions, rather than as demonstrated effects. The figures and testable predictions presented in later sections should therefore be interpreted as conceptual models and candidate experimental designs, not as summaries of proven therapeutic mechanisms.

This explicit separation is intended to reduce the risk of over-interpretation and to align the manuscript with its status as a Hypothesis article, whose primary aim is to structure future research questions rather than to report definitive mechanistic or clinical results.

## 3. Foundations of Neural Timing and Prefrontal Control in Epilepsy

Epilepsy is increasingly understood as a disorder of large-scale network dynamics rather than isolated hyperexcitable zones. Contemporary models conceptualize seizure generation and propagation as an emergent property of ictogenic networks—distributed cortical and subcortical circuits whose structural and functional properties determine how likely it is that ongoing activity will evolve into a seizure, rather than as isolated hyperexcitable foci [21,22]. Within these networks, prefrontal and fronto-parietal control systems play a crucial regulatory role, exerting top-down inhibition that constrains the spread of aberrant activity under physiological conditions [23].

### 3.1. Ictogenic Networks and Fronto-Interhemispheric Dysconnectivity

Recent network-based studies (EEG, MEG, fMRI) demonstrate that individuals with focal epilepsy often exhibit reduced frontal and interhemispheric functional connectivity, particularly within the anterior cingulate, dorsolateral prefrontal cortex, and supplementary motor areas [24,25]. Disruptions in these hubs weaken inhibitory control and increase vulnerability to runaway synchronization.

Multiple investigations conducted between 2021 and 2025 have highlighted:

- decreased interhemispheric coherence in the delta–theta range in frontal networks [24];
- impaired integrity of callosal pathways associated with seizure generalization risk [25];
- alterations in cross-frequency coupling between prefrontal and temporal cortices [26].

These findings suggest that interhemispheric coordination is not merely a marker of network health but plays an active role in regulating excitability and preventing pathological spread.

### 3.2. Temporal Dysregulation and Prefrontal Timing Networks

Beyond spatial networks, increasing evidence points to temporal dysregulation as a feature of epilepsy. The prefrontal cortex is central to human timing processes—interval estimation, temporal prediction, and sequencing—and relies on slow (0.8–1.5 Hz) intrinsic oscillations for temporal structuring of behavior [27].

Recent studies indicate that:

- patients with focal epilepsy show distortions in internal timing, including temporal compression and impaired synchronization to rhythmic cues [28];
- prefrontal timing networks exhibit reduced entrainment flexibility and diminished phase-locking to slow auditory rhythms [29];
- disruptions in slow oscillatory activity correlate with cognitive dyschronia and reduced attentional stability [30].

These findings are relevant because temporal structures act as regulators of network stability; disturbances in timing may lower the threshold for seizure spread.

### 3.3. Multisensory Integration and Rhythmic Entrainment

Auditory and somatosensory systems have robust capacities for neural entrainment, where external rhythmic stimulation induces phase alignment of endogenous oscillations. Between 2021 and 2025, converging evidence has shown that:

- auditory rhythmic cues modulate prefrontal oscillations in a frequency-specific manner [31];
- vibrotactile stimulation produces synchronized responses in sensorimotor and frontal cortices, engaging thalamo-cortical loops [32];
- combined auditory–tactile rhythmic stimulation enhances cross-modal integration and improves temporal prediction accuracy [33].

These mechanisms demonstrate that rhythmic sensory cues can act as external temporal references, influencing networks relevant to inhibitory control, attention, and large-scale coordination—domains altered in epilepsy.

### 3.4. Relevance to Epileptic Network Physiology

Together, disruptions in interhemispheric coordination, temporal processing, and entrainment susceptibility provide a mechanistic basis for exploring whether multisensory rhythmic stimulation might transiently modulate network properties relevant to seizure dynamics.

Importantly, these concepts do not imply therapeutic effects; instead, they highlight potential pathways through which non-invasive rhythmic cues may interact with neural systems already implicated in seizure generation and propagation.

Given that prefrontal timing networks interface with several known ictogenic hubs—including dorsolateral prefrontal, orbitofrontal, and anterior cingulate circuits—disturbances in slow temporal structure may lower the threshold for seizure spread by weakening top-down inhibitory control.

### 3.5. Integration of Recent Systems-Level Brain Research

Recent advances in systems neuroscience emphasize that brain disorders should be conceptualized not merely as localized circuit abnormalities but as disturbances of distributed, dynamic networks operating across multiple spatial and temporal scales.

Two recent comprehensive reviews on brain-related diseases [34,35] highlight the importance of cross-domain integration, including molecular, cellular, network, behavioral and computational perspectives. These works argue that pathological states often emerge from disruptions in large-scale network organization rather than isolated focal deficits. They also emphasize that mechanistic models must account for interactions between biological constraints, environmental modulation, and dynamic network reconfiguration.

The present conceptual framework aligns with this systems-level perspective by situating multisensory ERS not as a direct modulator of focal epileptic zones, but as a potential perturbation to distributed timing, attentional and prefrontal control networks. The VIW studies further underscore the value of integrating computational modeling, multimodal monitoring and mobile health technologies when examining complex dynamical processes in neurological disease—principles that directly inform Section 4 and Section 5 of this manuscript. In this context, ERS is framed as a controlled external perturbation that may help probe large-scale network properties rather than target single cortical regions.

## 4. Proposed Mechanistic Model of ERS in Focal Epilepsy

The following section outlines a hypothesis-driven mechanistic model describing how exogenous rhythmic stimulation (ERS) delivered through mobile multisensory cues may transiently influence cortical network dynamics relevant to focal epilepsy. The model integrates current knowledge on neural entrainment, prefrontal inhibitory control, interhemispheric coherence, and large-scale network stability, and is intended as a conceptual framework rather than a validated therapeutic mechanism.

The relevance of such distributed network interactions is supported by recent systems-level analyses of brain disorders [34,35], which emphasize that externally applied perturbations—whether sensory, cognitive, or neuromodulatory—tend to influence global network configurations rather than isolated cortical nodes. These studies reinforce the idea that ERS should be treated as a potential probe of large-scale network dynamics rather than a localized stimulation paradigm, consistent with the non-focal and multimodal nature of rhythmic sensory input.

### 4.1. ERS as an External Temporal Reference

Patients with focal epilepsy frequently demonstrate disturbances in slow oscillatory timing and reduced stability of prefrontal temporal networks [36]. Because auditory and vibrotactile rhythms in the low-frequency range (~1 Hz) reliably entrain large-scale cortical oscillations [37], ERS may provide an external temporal reference signal, compensating—at least transiently—for the instability of endogenous slow rhythms.

Such external cues can restore phase regularity in prefrontal circuits, potentially facilitating more coherent long-range communication. This mechanism is analogous to temporal realignment observed in auditory pacing paradigms in other neurological conditions [38].

### 4.2. Attentional Reallocation and Top-Down Network Engagement

Multisensory rhythmic cues engage dorsal and ventral attention networks and shift cognitive resources toward externally driven temporal structure [39]. This attentional reallocation may engage prefrontal inhibitory systems that are often weakened in epilepsy.

Increased engagement of the fronto-parietal control network may, in turn, transiently strengthen top-down suppression of cortical noise, which has been shown to modulate the stability of ictogenic networks [40].

This mechanism does not imply seizure prevention but may alter momentary susceptibility to disorganized network propagation.

### 4.3. Multisensory Rhythmic Entrainment and Sensorimotor Coupling

Simultaneous auditory, vibrotactile, and tapping stimulation facilitates multisensory entrainment, which is stronger than unimodal stimulation and results in enhanced synchronization across homologous cortical areas [41]. Sensorimotor coupling through rhythmic tapping additionally recruits premotor and supplementary motor areas, creating a distributed oscillatory scaffold.

In the context of focal epilepsy, where interhemispheric dysconnectivity is often pronounced [42], such multisensory inputs may temporarily promote cross-hemispheric coordination.

**Figure 1:**
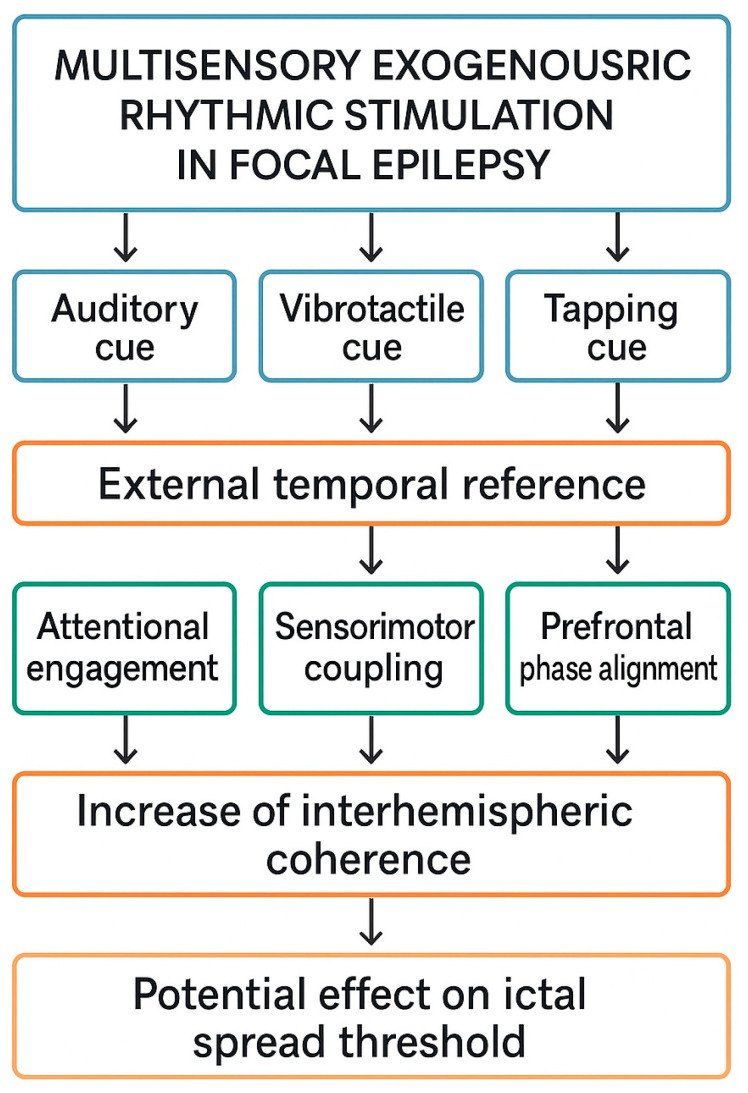
Conceptual mechanistic model of multisensory exogenous rhythmic stimulation (ERS) in focal epilepsy.

A schematic mechanistic framework summarizing the proposed multisensory ERS model is presented in Figure 1. The model integrates established principles of auditory and vibrotactile entrainment, prefrontal timing network dynamics, and interhemispheric coherence regulation, outlining how external rhythmic cues may transiently influence large-scale cortical network organization in focal epilepsy. Importantly, the framework is conceptual and does not imply therapeutic effectiveness; its purpose is to delineate plausible neurophysiological pathways that warrant empirical testing.

The diagram illustrates a hypothesis-driven framework describing how auditory, vibrotactile, and tapping rhythmic cues—delivered via mobile technologies—may act as an external temporal reference and interact with cortical networks implicated in focal epilepsy. Multimodal rhythmic input is proposed to (1) enhance attentional engagement, (2) induce sensorimotor coupling, and (3) promote prefrontal phase alignment through established entrainment mechanisms. These processes may transiently increase interhemispheric coherence within frontal networks, as observed in exploratory EEG studies, and thereby modulate large-scale network stability. The model is conceptual and does not imply clinical efficacy; it outlines hypothetical pathways requiring empirical validation in neurophysiological and behavioral studies.

### 4.4. Transient Modulation of Interhemispheric Frontal Coherence (Conceptual Interpretation)

Empirical studies of focal epilepsy consistently report alterations in interhemispheric coherence, particularly within prefrontal and anterior cingulate networks, although the direction and functional meaning of these abnormalities vary across syndromes, frequency bands, and behavioral states. In some patient groups, reduced frontal coherence has been associated with impaired top-down control and increased vulnerability to disorganized network propagation, whereas in others, excessive hypersynchrony may reflect pathological recruitment of large-scale cortico–subcortical loops. Because of this heterogeneity, coherence cannot be interpreted as a uniformly “beneficial” or “detrimental” marker.

Within this conceptual framework, the hypothesized effect of multisensory ERS is limited to the short-term modulation of band-limited frontal coherence, particularly in networks that show reduced baseline coupling. Such modulation is proposed as a candidate physiological marker of enhanced temporal alignment or attentional engagement, not as evidence of improved clinical network stability. Exploratory EEG observations from a small pilot study involving mobile ERS exposure suggested a transient increase in low-frequency interhemispheric coherence; however, these findings were not obtained under controlled conditions, did not undergo systematic correction for multiple comparisons, and cannot be generalized beyond the specific cohort studied.

Importantly, the conceptual model does not assume that coherence changes represent a direct mechanistic pathway to seizure modulation. Rather, coherence is treated as a proxy measure that may reflect shifts in large-scale network organization during rhythmic sensory input. This proxy status is emphasized because coherence can arise from multiple physiological sources—attention, sensory prediction, multisensory integration, or even volume conduction—and future studies must employ stricter controls (e.g., imaginary coherence, PLI, source reconstruction, sham stimulation) to isolate entrainment-specific contributions.

**Figure 2:**
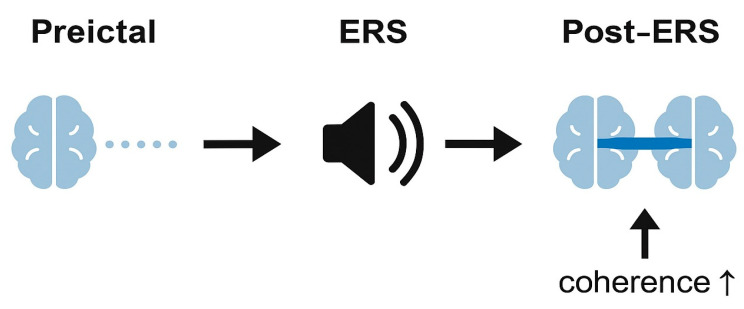
Hypothesized temporal effect of exogenous rhythmic stimulation (ERS) on frontal network coherence.

Figure 1 and Figure 2 illustrate these ideas in a schematic manner, depicting how rhythmic cues might transiently align prefrontal activity without implying causal or therapeutic effects. They should therefore be interpreted as hypothesis-generating diagrams, not as representations of established neurophysiology.

### 4.5. Conceptual Considerations Regarding Ictal Spread and Network Stability

Contemporary models of ictogenic networks emphasize that seizure initiation and propagation depend on the interaction between local excitability, large-scale connectivity, and state-dependent inhibitory control. Computational studies suggest that modulations in long-range connectivity—particularly within prefrontal control networks—may alter the ease with which focal activity recruits additional regions. These models, however, describe general network principles and do not provide evidence that sensory stimulation can directly influence seizure thresholds in humans.

In the present framework, the discussion of ictal spread is strictly hypothetical and serves only to outline how future mechanistic studies might investigate ERS-induced changes in large-scale dynamics. Specifically, if rhythmic cues were shown—under controlled conditions—to modulate frontal coherence or temporal alignment, such changes could be explored as candidate variables in computational simulations of ictogenic networks. These simulations could test whether small shifts in prefrontal coupling are theoretically capable of altering network resilience or the critical transitions associated with seizure spread.

Recent hybrid modeling approaches demonstrate how physics-based simulations can be combined with AI-driven inference to generate verifiable mechanistic predictions and to optimize device-level performance. For example, Pratticò et al. (2025) [43] integrated finite-element thermal modeling with deep-learning architectures (U-Net and MLP) to detect and classify thermal anomalies in biomedical electronic systems with high accuracy. Although the domain differs substantially from epilepsy, this study illustrates a generalizable methodological principle: biophysically grounded models can be coupled with AI-based prediction frameworks to bridge conceptual mechanisms and empirical validation. In future work, a similar hybrid strategy could be used to embed ERS-related neural-mass or network models into FEM-based simulations to formally test how slow rhythmic inputs might influence large-scale network stability under defined parameter regimes [44].

It is essential to emphasize that no empirical evidence currently demonstrates that the transient coherence changes observed in exploratory ERS studies affect seizure susceptibility, onset, or propagation. The present article does not propose ERS as a therapeutic intervention and does not claim that sensory-driven modulation of frontal coherence leads to improved network stability. Instead, the conceptual model highlights mechanistic pathways that remain purely speculative until validated by (I) computational modeling, (II) controlled neurophysiological experiments, (III) systematic safety evaluations, and (IV) clinical studies conducted under rigorous monitoring.

Accordingly, any mention of “network stabilization” in this manuscript should be understood as a theoretical construct, not a confirmed physiological or clinical effect.

## 5. Exploratory Implementation Example: Mobile ERS Application Model

To illustrate the practical implementation of multisensory exogenous rhythmic stimulation (ERS) within a real-world mobile environment, we describe an example of a mobile ERS system. This section does not present EpiTapp^®^ as a therapeutic or clinically validated intervention. Instead, it serves as a technological demonstration of how auditory, vibrotactile, and motor-synchronization cues can be integrated into a mobile platform for experimental neuromodulation research.

### 5.1. Multimodal ERS Delivery Through Mobile Devices

Modern smartphones and wearable devices incorporate high-precision vibration motors, multichannel audio output, inertial sensors, and touch interfaces. These features allow the delivery of coordinated multisensory rhythmic cues with controlled frequency, amplitude, and timing. Such integrated platforms have been used experimentally in the study of attention, pacing, and entrainment phenomena in neurological populations [45].

A mobile ERS system typically includes:

- Auditory rhythmic pulses (e.g., 0.8–1.2 Hz), delivered through headphones or speakers;
- Vibrotactile cues synchronized to auditory timing;
- Manual tapping input, enabling sensorimotor coupling and user-driven phase alignment;
- Logging functions to record timing, duration, and user response patterns.

These features facilitate controlled, reproducible stimulation in naturalistic settings without external hardware.

### 5.2. ERS Timing Architecture and User Interaction

ERS timing engines can synchronize multimodal outputs with millisecond accuracy using built-in OS-level audio and haptic APIs. The user interacts with the system via:

- tactile feedback loops,
- tapping synchronization tasks,
- optional self-reporting modules.

This closed-loop user-driven interaction aims to promote engagement of attentional and prefrontal timing networks—mechanisms compatible with the conceptual framework presented earlier.

### 5.3. EEG-Based Exploratory Findings (Pilot Study): Methods, Results, and Limitations

The exploratory EEG findings referenced in this manuscript originate from a previously conducted pilot study of mobile ERS exposure in adults with focal epilepsy [20]. The study was not designed as a controlled experiment, did not include a sham or non-rhythmic condition, and therefore its results should be interpreted solely as preliminary observations illustrating the feasibility of integrating mobile rhythmic stimulation with EEG recording. For transparency and to clarify the scope of these findings, key methodological parameters and limitations are summarized below.

Participants and Recording Setup.

The pilot included a small convenience sample of adults diagnosed with structural focal epilepsy (*N* = 12; ages approximately 20–45 years), recruited from an outpatient neurology clinic. All participants had stable antiseizure medication regimens, and none had photosensitivity or a history of musicogenic or pattern-triggered seizures. EEG was recorded using a standard 10–20 montage with a sampling rate of 500 Hz (hardware specifications comparable to routine clinical EEG systems). Data quality and electrode placement followed standard clinical practice.

ERS Parameters.

In the pilot implementation, auditory stimuli consisted of brief (tens of milliseconds) broadband pulses presented at a comfortable listening level approximately once per second (~1 Hz). Vibrotactile cues were delivered as short vibration bursts of comparable duration via the smartphone motor, time-locked to the auditory pulses. The effective duty cycle of stimulation was therefore low, with short pulses separated by long interpulse intervals without stimulation. Temporal accuracy was verified at the software level using internal timing logs, confirming that inter-pulse intervals clustered around the nominal 1 s period with only small jitter on the order of tens of milliseconds. These parameters were chosen to ensure clear perceptibility while minimizing continuous sensory load and energy consumption.

Preprocessing Pipeline.

EEG data were high-pass filtered at 1 Hz, low-pass filtered at 40 Hz, and subjected to 50 Hz notch filtering. Artifacts due to eye blinks and muscle activity were reduced through a combination of manual inspection and ICA-based component rejection. Analyses focused on homologous frontal derivations (e.g., F3–F4, Fp1–Fp2), given the conceptual relevance of prefrontal coherence to the proposed mechanistic framework.

Coherence Metrics and Statistical Approach.

Interhemispheric coherence was computed using magnitude-squared coherence (MSC) within conventional frequency bands. Because the pilot study lacked a formal experimental design, only paired comparisons were performed between a short resting baseline and a brief ERS exposure period (~1 Hz multisensory pacing). Effect sizes (Cohen’s d) were estimated for descriptive purposes. Key descriptive statistics for frontal interhemispheric coherence are summarized in Table 1. No correction for multiple comparisons was applied at the time of the original analysis; therefore, any band-specific findings must be considered exploratory and potentially inflated by Type I error. In future studies, correction via false discovery rate (FDR) or cluster-based permutation testing will be required.

Summary of Observations.

A subset of participants showed modest increases in low-frequency frontal coherence during ERS exposure relative to baseline. These signals were variable across individuals, and no consistent pattern was observed outside the low-frequency range. Because the study did not include a sham condition, these changes may reflect attention, expectancy, or sensory engagement rather than entrainment-related mechanisms. The amplitude of coherence modulation varied across participants, suggesting syndrome- and network-specific differences that require systematic investigation.

Interpretational Boundaries.

These exploratory observations should not be interpreted as evidence that ERS modulates seizure susceptibility, improves network stability, or produces clinically meaningful effects. The absence of control conditions, small sample size, lack of source-space analysis, and uncorrected multiple comparisons preclude mechanistic or causal inference. Moreover, coherence increases may arise from non-neural factors such as residual volume conduction or movement-related contamination. Future work must therefore incorporate imaginary coherence or phase-lag index (PLI), motion sensor data, and rigorous sham stimulation blocks to isolate true entrainment effects.

Role of the Pilot Study in the Present Article.

The pilot findings are included only to illustrate that mobile delivery of rhythmic cues is feasible in this population and to motivate further hypothesis-driven research. They do not serve as empirical validation of the mechanistic model proposed in Section 4. All mechanistic claims in this manuscript remain conceptual, and the pilot results provide no evidence regarding seizure control or therapeutic potential.

Overall, this pilot serves as an illustrative example rather than a basis for generalization. Robust mechanistic conclusions will require controlled studies with larger cohorts, standardized coherence metrics, sham conditions, and a priori statistical correction.

These values represent approximate descriptive summaries extracted from averaged frontal channel pairs (e.g., F3–F4) in the pilot dataset. Given the exploratory nature of the recordings, small sample size, and absence of sham or counterbalanced conditions, these metrics should not be interpreted as evidence of a stimulation effect. The table is provided solely to increase methodological transparency as requested by reviewers.

### 5.4. Alignment with the Mechanistic Model

Within the conceptual framework described in Section 4, the mobile ERS implementation illustrates how:

- auditory + vibrotactile + tapping cues can act as an external temporal reference,
- multisensory stimulation can reinforce attentional engagement and sensorimotor coupling,
- rhythmic inputs may induce phase alignment across prefrontal networks,
- momentary increases in interhemispheric coherence may emerge as a measurable physiological correlate.

These observations provide a technological example consistent with the hypothesized mechanisms but do not indicate clinical benefit.

### 5.5. Limitations of the Implementation Example

- No clinical outcomes or seizure metrics were assessed.
- The system is not a registered medical device.
- Findings require replication in larger, controlled neurophysiological studies.
- The example serves strictly as an illustration, not as evidence of therapeutic effect.

### 5.6. Hardware–Software Co-Design and Engineering Considerations for Mobile ERS

Implementing multisensory ERS on mobile platforms requires coordinated audio–haptic timing, energy-efficient actuator control, and predictable latency across consumer-grade hardware. While the present study does not evaluate device performance empirically, several engineering principles are relevant for future development and were informed by recent work on embedded intelligence and energy-aware neuromodulation systems [46].

A relevant engineering analogy comes from recent work on embedded intelligent systems. Bibbò et al. (2025) [46] demonstrated how energy-aware architectures can integrate renewable energy management, embedded AI modules, and real-time optimization strategies in mobile and resource-constrained platforms. Although their system targets UAV logistics rather than biomedical applications, the underlying principles—balancing actuator load, optimizing duty cycles, and coordinating sensing with computation—translate directly to future ERS implementations. These frameworks highlight how mobile neuromodulation tools may require joint optimization of timing accuracy, actuator efficiency, and continuous monitoring under real hardware constraints.

Timing accuracy and multisensory synchronization.

Multisensory ERS relies on stable phase alignment between auditory pulses, vibrotactile cues, and optional tapping-based sensorimotor feedback. Mobile operating systems typically introduce timing jitter due to audio buffer scheduling, vibration motor activation delays, and background process load. To ensure reliable multisensory synchronization, ERS systems may require:

(I) pre-buffered audio playback with fixed latency pathways;
(II) low-level haptic timing APIs capable of sub-20 ms accuracy;
(III) real-time timestamping of user tapping events to align endogenous responses with external rhythms.

Although absolute phase precision on consumer devices remains limited compared to laboratory equipment, engineering strategies such as clock drift compensation and dynamic latency calibration can maintain acceptable entrainment fidelity for low-frequency stimulation (~1 Hz).

Energy consumption and thermal constraints.

Continuous vibrotactile stimulation can impose nontrivial energy demands. Insights from energy-aware wearable design [46] suggest that long-duration ERS protocols will require:

(I) optimized duty cycles (e.g., short haptic bursts rather than continuous vibration);
(II) amplitude modulation strategies that reduce actuator load without degrading perceptibility;
(III) adaptive power management algorithms that minimize battery drain during combined audio–haptic output.

These considerations are particularly relevant for closed-loop ERS implementations or ecological field studies, where extended runtime is necessary.

Latency constraints and real-time feedback.

For ERS paradigms that incorporate tapping or movement-based sensorimotor coupling, round-trip latency (stimulus→user response→device detection→next stimulus adjustment) must remain predictable. High latency variability may disrupt alignment between external rhythmic cues and neural prediction processes. Future implementations should therefore use inertial sensors with consistent sampling rates, and on-device processing pipelines that avoid cloud-based delays. Latency thresholds of <100 ms are likely adequate for low-frequency entrainment but require empirical validation.

Monitoring continuity and data integrity.

Mobile ERS systems that log user behavior, tapping accuracy, or device interaction must ensure stable data capture during stimulation. Sensor dropout, frame loss, and OS-imposed app suspension are common challenges in mobile environments. Following principles outlined for embedded neuromodulation and energy-constrained systems [46], ERS applications may need:

(I) redundant buffering of sensor streams;
(II) integrity checks for timestamp continuity;
(III) local storage protocols with asynchronous sync to remote servers when available.

These measures reduce data loss and enable reliable reconstruction of stimulation–behavior coupling during analysis.

Engineering–physiological alignment.

Importantly, these hardware/software considerations are not mere technical details but shape the physiological interpretability of ERS. Latency jitter affects entrainment strength; actuator amplitude affects somatosensory recruitment; energy constraints limit session duration; and data continuity determines whether behavioral and EEG measurements can be meaningfully correlated. As shown in engineering frameworks integrating neuromodulation with embedded intelligence [46], co-design of hardware and physiological goals is essential for translating conceptual models into experimentally testable systems.

The present article does not evaluate these engineering factors experimentally; instead, they are outlined to contextualize the constraints and requirements for future mobile ERS implementations. Any translational work must explicitly address timing accuracy, actuator performance, energy consumption, latency stability, and data integrity to ensure methodological rigor.

## 6. Limitations

This work presents a conceptual and hypothesis-driven model of multisensory exogenous rhythmic stimulation (ERS) in focal epilepsy. Several important limitations should be acknowledged.

First, the proposed mechanisms are extrapolated from studies of auditory and vibrotactile entrainment, multisensory integration, and prefrontal timing networks in neurological (e.g., Parkinson’s disease, stroke rehabilitation, movement disorders) and healthy populations. Only limited and heterogeneous evidence exists regarding sensory-driven entrainment specifically in epilepsy, and available findings are primarily exploratory, derived from small cohorts, and rarely include seizure-related outcomes. As a result, the present framework should be viewed as a transfer of general principles of neural timing and network control to the context of ictogenic networks, rather than as evidence-based support for disease-specific therapeutic efficacy.

Second, the mechanistic interpretations rely on indirect markers—such as interhemispheric frontal coherence—which reflect large-scale network dynamics but do not directly index ictogenic processes. No causal relationship between ERS exposure and seizure susceptibility can be inferred from current evidence.

Third, the conceptual framework incorporates data from heterogeneous methodologies (EEG studies, behavioral entrainment paradigms, and neuromodulation research). These disciplines differ in spatial and temporal resolution, making cross-domain integration inherently speculative.

Fourth, the mobile ERS implementation example is provided for illustrative purposes only. It is not a certified medical device, and available findings do not include clinical outcomes such as seizure frequency, severity, or functional measures.

Finally, the model does not address inter-individual variability in epilepsy etiology, network topology, comorbidities, medication effects, or behavioral factors, all of which may profoundly influence responsiveness to external rhythmic cues.

Overall, this article outlines theoretical pathways that require validation through controlled neurophysiological experiments, mechanistic studies, and eventually carefully designed clinical trials.

### Safety and Data Privacy Considerations

Sensory stimulation paradigms require explicit safety considerations in epilepsy research, even when they do not involve direct electrical currents. Although the low-frequency rhythmic cues proposed in this framework differ fundamentally from known seizure-provoking stimuli (e.g., high-contrast photic flicker or complex musical structures), patterned auditory and vibrotactile inputs may still interact with ictogenic networks in unpredictable ways. The present conceptual model therefore does not assume safety or tolerability, and all potential risks must be addressed in future feasibility studies before any clinical conclusions can be drawn.

Safety considerations related to seizure provocation.

Unlike classical photosensitive triggers—typically occurring at 10–30 Hz with high luminance contrast—or musicogenic triggers involving emotionally salient or structurally complex auditory sequences, the multisensory ERS paradigm described here relies on low-frequency (~1 Hz) auditory and vibrotactile pulses with minimal spectral complexity. These features make seizure provocation unlikely based on current mechanistic understanding. However, because rare cases of stimulus-induced seizures have been documented in epilepsy (e.g., musicogenic epilepsy, pattern sensitivity), any rhythmic sensory protocol requires systematic safety monitoring. Prior to formal experimentation, individuals with known photosensitivity, pattern sensitivity, or musicogenic seizure triggers should be excluded.

Monitoring of epileptiform activity and adverse events in future studies.

The exploratory pilot referenced in this manuscript did not include real-time seizure monitoring or systematic adverse event reporting; thus, no claims regarding safety can be made. Future studies must incorporate:

(I) continuous EEG monitoring during ERS exposure;
(II) standardized scoring of interictal epileptiform discharges and state changes;
(III) structured adverse-event logs documenting auras, discomfort, anxiety, and seizure-related symptoms;
(IV) pre-defined stopping criteria (e.g., increase in epileptiform activity, participant discomfort, or sustained autonomic changes).

These measures are essential to ensure participant safety and to determine whether rhythmic sensory cues influence ictogenic markers or behavioral state.

Principles for designing safe experimental protocols.

Early studies should employ conservative stimulation parameters, beginning with short trial blocks (e.g., 10–20 s), low stimulus amplitude, and extended rest intervals. Participants should be supervised by clinicians trained in seizure recognition, and sessions should be terminated immediately if seizure warning signs occur. Sham and non-rhythmic control conditions must be included to differentiate entrainment-specific effects from nonspecific factors such as arousal or novelty. For populations at elevated risk (e.g., juvenile myoclonic epilepsy, individuals with generalization-prone temporal lobe epilepsy), safety protocols should be even more restrictive or exclude ERS exposure entirely until preliminary evidence of tolerability is established.

Data privacy considerations for mobile ERS systems.

If ERS paradigms are delivered through consumer devices (e.g., smartphones or wearables), data security becomes critical. Timing logs, usage metadata, and potentially sensitive clinical information may be stored locally or transmitted to external servers. Future implementations must therefore:

(I) minimize collection of identifiable data;
(II) ensure end-to-end encryption for data transmission;
(III) obtain explicit informed consent describing storage and processing of usage metrics;
(IV) comply with relevant data protection regulations (e.g., GDPR-aligned requirements, regional health-data policies).

Scope of the present article.

The conceptual mechanistic framework presented here does not evaluate clinical safety, does not endorse ERS as a therapeutic tool, and does not imply that multisensory rhythmic cues reduce seizure risk. All discussion related to seizure propagation, network stability, or ictogenic thresholds is purely hypothetical. Any future clinical translation will depend on rigorous safety assessments, controlled neurophysiological monitoring, and formal regulatory evaluation.

## 7. Future Directions

Future research should aim to translate the conceptual model of multisensory ERS into empirically testable frameworks.

1. Controlled neurophysiological validation.
  Well-designed EEG and MEG studies are needed to quantify ERS-induced changes in phase synchronization, interhemispheric coupling, and low-frequency oscillatory stability in patients with focal epilepsy. Such work should determine whether observed coherence shifts are reproducible, frequency-specific, and mechanistically linked to attentional and multisensory processes.
2. Characterization of individual variability.
  Future studies should investigate how ERS responses vary across epilepsy syndromes, lesion types, comorbidities, cognitive profiles, and medication regimens. Personalized entrainment models may be required to match stimulus parameters (frequency, modality, intensity) to individual network architectures.
3. Integration with wearable and predictive systems.
  ERS could be combined with wearable seizure detection or forecasting algorithms to explore timing-specific or state-dependent stimulation paradigms. Real-time adaptive ERS, triggered by physiological risk markers, represents a potential direction for digital neuromodulation research.
4. Mechanistic modeling and computational simulation.
  Computational models of ictogenic networks could be used to simulate how multisensory rhythmic inputs influence network stability, inhibitory control, and thresholds for ictal spread, thereby refining mechanistic hypotheses.
5. Preliminary feasibility and safety studies.
  Before evaluating clinical outcomes, early pilot studies should examine acceptability, tolerability, user engagement, and cognitive/behavioral correlates of ERS in naturalistic settings.
6. Ethical, regulatory, and translational considerations.

Given the sensory-based nature of ERS, future research must address data privacy, safety standards, and regulatory classification for digital neuromodulation tools, ensuring responsible integration into clinical practice.

Taken together, these directions outline a roadmap for transitioning ERS from a theoretical construct to an experimentally grounded approach within digital neuromodulation.

### Testable Predictions

The conceptual model proposed in this article is intended to generate empirically testable hypotheses about how multisensory ERS may transiently influence large-scale network dynamics in focal epilepsy. The following predictions are formulated with explicit parameters, measurable outcomes, and modality-specific contrasts, to facilitate rigorous experimental testing. None of these predictions should be interpreted as implying therapeutic benefit; rather, they outline candidate physiological effects that can be quantified in controlled studies.

Prediction 1: Frequency-specific modulation of frontal coherence.

Low-frequency multisensory ERS is hypothesized to produce a measurable increase in frontal interhemispheric coherence within the 0.8–1.2 Hz range relative to resting baseline. This effect should be observable using magnitude-squared coherence, imaginary coherence, or phase-lag index (PLI), and should exceed a predefined threshold (e.g., ≥0.3 SD above individual baseline variability). Frequency sweep designs (e.g., 0.5, 1.0, 2.0, and 4.0 Hz) will allow testing whether ~1 Hz stimulation produces the strongest coherence modulation, as suggested by models of prefrontal slow oscillatory timing.

Prediction 2: Multisensory ERS will show stronger network effects than unimodal stimulation.

When auditory-only, vibrotactile-only, and combined auditory–vibrotactile stimulation are presented in randomized blocks, multisensory ERS is expected to yield larger coherence changes (or greater phase-locking consistency) than unimodal stimulation. Effects should be quantified with within-subject contrasts (multisensory > auditory; multisensory > vibrotactile) using normalized coherence or phase-locking values, while controlling for volume conduction via imaginary metrics.

Prediction 3: Individual responsiveness will correlate with baseline temporal processing.

Individuals exhibiting greater baseline deficits in temporal estimation, temporal compression, or reduced phase-locking to external rhythms are predicted to show stronger ERS-induced modulation of frontal network markers. This correlation should be evaluated using standardized behavioral measures of interval timing and cross-trial phase consistency in EEG.

Prediction 4: ERS-induced changes will be transient and state-dependent.

Any modulation of coherence or cross-hemispheric coupling is expected to revert to baseline levels shortly after stimulation (e.g., within 30–120 s). This predicts a time-limited effect, testable through post-stimulation epochs divided into early (0–30 s), intermediate (30–60 s), and late (60–120 s) windows.

Prediction 5: No changes should appear during sham or non-rhythmic control conditions.

When stimulation is replaced by non-periodic cues with matched sensory intensity (sham condition), no systematic change in coherence or phase-locking is expected. This contrast (ERS rhythmic > non-rhythmic sham) is critical to dissociate entrainment-related mechanisms from attentional or expectancy-based effects.

Prediction 6: Computational simulations will predict directionally consistent changes.

Neural mass or reduced network models incorporating an external periodic drive at ~1 Hz are expected to show small but directionally consistent shifts in long-range coupling parameters. These shifts may approximate the empirical coherence changes observed during ERS, thus providing a mechanistic bridge between empirical and modeling domains. However, such simulations do not imply causal influence on ictal thresholds.

Taken together, these predictions provide operationalized, parameter-defined hypotheses that can be tested in controlled neurophysiological and computational studies. Their purpose is to refine the conceptual framework presented in this article, not to infer clinical effects or therapeutic implications.

## 8. Conclusions

This article proposes a hypothesis-driven mechanistic framework describing how multisensory exogenous rhythmic stimulation (ERS)—delivered through auditory, vibrotactile, and sensorimotor cues—may interact with neural timing systems and large-scale cortical networks relevant to focal epilepsy. The model integrates evidence from entrainment research, prefrontal control theories, multisensory integration, and exploratory neurophysiological findings, including preliminary increases in interhemispheric frontal coherence observed during mobile ERS exposure.

ERS is presented not as a therapeutic intervention, but as a conceptual tool for investigating how external rhythmic structure may transiently influence network synchronization and attentional control. The proposed mechanisms remain speculative and require validation through controlled neurophysiological experiments, computational modeling, and carefully designed clinical studies.

By outlining potential pathways through which multisensory rhythmic cues could modulate large-scale network dynamics, this work establishes a theoretical foundation for future research in digital neuromodulation and sensory-based interventions in epilepsy. The framework may guide the development of next-generation experimental tools aimed at probing network stability, temporal processing, and adaptive control in drug-resistant epilepsy.

## Figures and Tables

**Table 1:** Approximate descriptive coherence values during baseline and ERS exposure in the exploratory pilot study. Values reflect preliminary estimates and are not intended for inferential interpretation.

Frequency Band	Baseline Coherence (Mean ± SD)	ERS Coherence (Mean ± SD)	*p*-Value	Cohen’s d
Delta (1–4 Hz)	0.28 ± 0.09	0.33 ± 0.10	n/a	n/a
Theta (4–8 Hz)	0.32 ± 0.11	0.36 ± 0.12	n/a	n/a
Alpha (8–12 Hz)	0.30 ± 0.08	0.31 ± 0.08	n/a	n/a

## Data Availability

No new data were created or analyzed in this study. Data sharing is not applicable to this article.
